# Death of Henri F. Formad

**Published:** 1892-07

**Authors:** 


### NECROLOGY.

### HENRI F. FORMAD.

Dr. Henri F. Formad, the eminent pathologist and microscopist, died suddenly June 6th at the home of his sister, Dr. Marie Formad, 1003 North Sixth street, Philadelphia. Dr. Formad had always enjoyed the most perfect health until some months since, when he suffered from an attack of blood poisoning. A few days before his death he was seized with an attack of acute gastro enteritis, which rapidly brought a fatal termination.

While Dr. Formad was not a veterinarian, he has been so closely associated with veterinarians that he was well known to the profession, and his loss will be acutely felt and sincerely mourned by all. His removal from scientific labors in veterinary pathology and his teaching of veterinary students at the University of Pennsylvania will be an irreparable loss to the profession at large, and his removal from his friends will leave a sad mourning for a kindly, agreeable companion.

Henri F. Formad was born March 10th, 1847, in Schelezno-wodsk, Province of Caukasus, Russia. His father was a merchant. who afterwards removed to Odessa. In his native city he was educated and graduated in medicine.

When a young man he was serving as an assistant surgeon in a Russian regiment, of which his uncle was colonel, when, with several others, he was arrested, charged with Nihilistic proclivities, and was sentenced to be shot at day-break, but escaped and reached Germany.

He graduated from the Heidelberg University. While occupying a chair there temporarily he came to America, and, taking a course at the University of Pennsylvania, graduated in 1877. He practiced medicine in West Philadelphia for two years, but always preferred the work of the labaratory and lecture-room to that of a practitioner, and soon his public positions demanded and received all of his time. He was appointed Demonstrator of Pathology in the Medical Department of the University of Pennsylvania, Pathologist to the City Hospital, and in 1884 succeeded Dr. Huidekoper as Coroner Physician of Philadelphia, which position he held until December, 1891.

Dr. Formad was an active member of the College of Physicians of Philadelphia, the Pathological Society, the Keystone Veterinary Medical Society and numerous other scientific associations. He was a member of the United States Commission for the Investigation of Diphtheria. From 1883 to 1886 he was the Miiter Lecturer, College of Physicians, Philadelphia.

Dr. Formad was widely known through his teaching, his writings and his positions as expert, as an authority upon microscopy, pathology and medical jurisprudence.

Dr. Formad was a generous man, and like most experts a man of pronounced ideas. He was a linguist of rare ability, speaking French, Russian, German, Greek and Latin.

During his term as Coroner's Physician he made two trips to Europe, both times as a delegate to the International Medical Congress. He took advantage of the opportunity to study in Vienna, Paris and Berlin. His last trip abroad was in 1890.

Dr. Formad for several years was preparing a work on microscopy, which was to be profusely illustrated. Much of the work is in type, and hundreds of cuts have been prepared for the book.

He held to the tenets of the Greek Church. In politics he was an active and pronounced Republican. He was a member of the Order of Sparta, of the Ancient Order of United Workmen, and of many social clubs, especially among the Germans. The readers of the JOURNAL will remember his valuable contribution upon Mammalian Blood.

